# High-Throughput Genomics Identify Novel *FBN1/2* Variants in Severe Neonatal Marfan Syndrome and Congenital Heart Defects

**DOI:** 10.3390/ijms25105469

**Published:** 2024-05-17

**Authors:** Gloria K. E. Zodanu, John H. Hwang, Zubin Mehta, Carlos Sisniega, Alexander Barsegian, Xuedong Kang, Reshma Biniwale, Ming-Sing Si, Gary M. Satou, Nancy Halnon, UCLA Congenital Heart Defect BioCore Faculty, Wayne W. Grody, Glen S. Van Arsdell, Stanley F. Nelson, Marlin Touma

**Affiliations:** 1 Neonatal Congenital Heart Laboratory, Department of Pediatrics, David Geffen School of Medicine, University of California, Los Angeles, CA 90095, USA; gzodanu@mednet.ucla.edu (G.K.E.Z.); johnhwang@mednet.ucla.edu (J.H.H.); zmehta7@gmail.com (Z.M.); csisniega@mednet.ucla.edu (C.S.); barsegian88@gmail.com (A.B.); xkang@mednet.ucla.edu (X.K.);; 2 Department of Pediatrics, David Geffen School of Medicine, University of California, Los Angeles, CA 90095, USA; rbiniwale@mednet.ucla.edu (R.B.); gsatou@mednet.ucla.edu (G.M.S.); nhalnon@mednet.ucla.edu (N.H.); wgrody@mednet.ucla.edu (W.W.G.); gvanarsdell@mednet.ucla.edu (G.S.V.A.); snelson@mednet.ucla.edu (S.F.N.); 3 Department of Surgery, David Geffen School of Medicine, University of California, Los Angeles, CA 90095, USA; msi@mednet.ucla.edu; 4 Department of Pathology and Laboratory Medicine, David Geffen School of Medicine, University of California, Los Angeles, CA 90095, USA; 5 Department of Human Genetics, David Geffen School of Medicine, University of California, Los Angeles, CA 90095, USA; 6 Molecular Biology Institute, University of California, Los Angeles, CA 90095, USA; 7 Children’s Discovery and Innovation Institute, University of California, Los Angeles, CA 90095, USA; 8 Eli and Edyth Broad Stem Cell Research Center, University of California, Los Angeles, CA 90095, USA; 9 Cardiovascular Research Laboratories, David Geffen School of Medicine, University of California, Los Angeles, CA 90095, USA

**Keywords:** Marfan syndrome (MFS), neonatal Marfan syndrome (nMFS), congenital contractural arachnodactyly (CAA), dextro-transposition of the great arteries (D-TGA), whole-exome sequencing (WES), whole-genome SNP microarray

## Abstract

Fibrillin-1 and fibrillin-2, encoded by *FBN1* and *FBN2*, respectively, play significant roles in elastic fiber assembly, with pathogenic variants causing a diverse group of connective tissue disorders such as Marfan syndrome (MFS) and congenital contractural arachnodactyly (CCD). Different genomic variations may lead to heterogeneous phenotypic features and functional consequences. Recent high-throughput sequencing modalities have allowed detection of novel variants that may guide the care for patients and inform the genetic counseling for their families. We performed clinical phenotyping for two newborn infants with complex congenital heart defects. For genetic investigations, we employed next-generation sequencing strategies including whole-genome Single-Nucleotide Polymorphism (SNP) microarray for infant A with valvular insufficiency, aortic sinus dilatation, hydronephrosis, and dysmorphic features, and Trio whole-exome sequencing (WES) for infant B with dextro-transposition of the great arteries (D-TGA) and both parents. Infant A is a term male with neonatal marfanoid features, left-sided hydronephrosis, and complex congenital heart defects including tricuspid regurgitation, aortic sinus dilatation, patent foramen ovale, patent ductus arteriosus, mitral regurgitation, tricuspid regurgitation, aortic regurgitation, and pulmonary sinus dilatation. He developed severe persistent pulmonary hypertension and worsening acute hypercapnic hypoxemic respiratory failure, and subsequently expired on day of life (DOL) 10 after compassionate extubation. Cytogenomic whole-genome SNP microarray analysis revealed a deletion within the *FBN1* gene spanning exons 7–30, which overlapped with the exon deletion hotspot region associated with neonatal Marfan syndrome. Infant B is a term male prenatally diagnosed with isolated D-TGA. He required balloon atrial septostomy on DOL 0 and subsequent atrial switch operation, atrial septal defect repair, and patent ductus arteriosus ligation on DOL 5. Trio-WES revealed compound heterozygous c.518C>T and c.8230T>G variants in the *FBN2* gene. Zygosity analysis confirmed each of the variants was inherited from one of the parents who were healthy heterozygous carriers. Since his cardiac repair at birth, he has been growing and developing well without any further hospitalization. Our study highlights novel *FBN1/FBN2* variants and signifies the phenotype–genotype association in two infants affected with complex congenital heart defects with and without dysmorphic features. These findings speak to the importance of next-generation high-throughput genomics for novel variant detection and the phenotypic variability associated with *FBN1/FBN2* variants, particularly in the neonatal period, which may significantly impact clinical care and family counseling.

## 1. Introduction

Fibrillins are large cysteine-rich glycoproteins that are major constituents of the extracellular matrix (ECM) that merge into microfibrils [1]. The fibrillin microfibrils are extensible polymers that provide connective tissue elasticity and are extensively spread in tissues and organs. They serve as templates for elastin deposition during elastic fiber formation and are essential for maintaining the integrity of tissues such as blood vessels, lungs, skin, and ocular ligaments [1,2,3]. The presence of a small proline-rich domain in fibrillin-1 that is replaced by a glycine-rich domain in fibrillin-2 is the main difference between fibrillin-1 and fibrillin-2 [4]. Nevertheless, the significance of these domains in matrix assembly is obscure and each fibrillin protein is encoded by a specific gene [4]. The proteins encoded by the two fibrillin genes, *FBN1* and *FBN2*, respectively, share 71.2% similarity but may play distinct significant roles in the formation of the ECM that may underlie varying phenotypic expressions (Table 1) [5,6].

**Figure 1:**
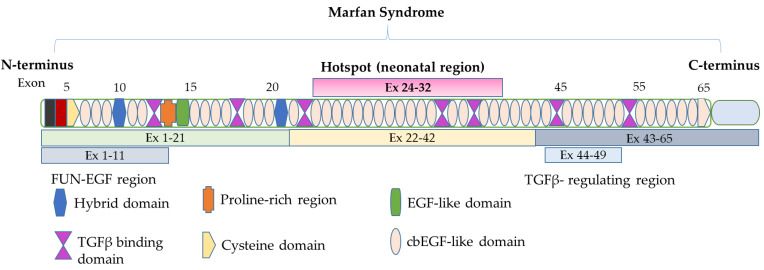
Schematic illustration of *FBN1* and protein structure domains. The *FBN1* gene is located on chromosome 15q21.1. The coding sequence consists of 65 exons. The hotspot neonatal region encodes a series of cbEGF-like domains. The cbEGF-like domain: homologous to calcium-binding epidermal growth factor; TGFβ-like domain: homologous to those in latent transforming growth factor beta; EGF-like domain: homologous to epidermal growth factor.

The gene encoding fibrillin-1 (*FBN1*: MIM:134797) encompasses 65 exons covering 200 kb of genomic DNA which is located on chromosome 15q21.1. Fibrillin-1 contains 47 motifs similar to the human epidermal growth factor (EGF) [7]. A total of 43 of these 47 EGF-like motifs present a consensus sequence for calcium binding (cbEGF) [8]. Each motif is composed of six cysteine residues that form disulfide bonds between C1 and C3, C2 and C4, and C5 and C6 which provides stability for its three-dimensional structure [7,9] (Figure 1). The calcium binding to cbEGF domains offers fibrillin-1 enhanced stability, protection against proteolysis, and structural elements for communication with numerous constituents of the ECM [7,9]. Wide stretches of these calcium-binding EGF-like motifs are disrupted by some important domains comprising seven of these motifs, with similarity to the latent transforming growth factor β1 binding protein (LTBP) [10]. 

Pathogenic variants in *FBN1* impair the formation of microfibrils, leading to the production of abnormal connective tissues, resulting in Marfan syndrome (MFS) (MIM:154700) [7,8,9], the most common inherited disease caused by *FBN1* pathogenic variants (Table 1) [11].

**Figure 2:**
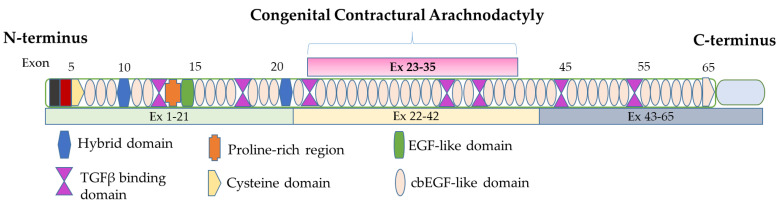
Schematic illustration of *FBN2* and protein structure domains. The *FBN2* gene is located on chromosome 5q23.3. The coding sequence consists of over 65 exons. The congenital contractural arachnodactyly region encodes a series of cbEGF-like and TGFβ-like domains.

The *FBN2* gene located on chromosome 5q23-3, spanning more than 28 kb and 65 exons, was identified while cloning the *FBN1* locus situated in 15q15-21.3 [6], and encodes fibrillin-2, a 2912 amino acid-containing protein with five different structural domains and length similar to fibrillin-1 [12,13]. The main structural region comprises 41 calcium binding-epidermal growth factor (cbEGF)-like domains [13] (Figure 2). Fibrillin-2 is expressed earlier in development and is constrained to bone and cartilage matrices in adult tissues and is essential in elastic fiber formation [3,5]. The gene *FBN2* has high similarity to *FBN1*; as a result, pathogenic variants in *FBN2* have been reported to cause a phenotypically related disorder termed congenital contractual arachnodactyly (CCA) (MIM:121050) (Table 1), which also presents with Marfanoid body habitus and arachnodactyly [14,15,16]. Due to the phenotypic similarities of CCA and MFS, establishing the incidence and prevalence has proved futile [17]. Herein we delineate the phenotypic features and genetic variants of two cases of fibrillinopathy associated with novel variants in *FBN1* and *FBN2*.

## 2. Results

### 2.1. History and Clinical Course

#### 2.1.1. Case A

**Figure 3:**
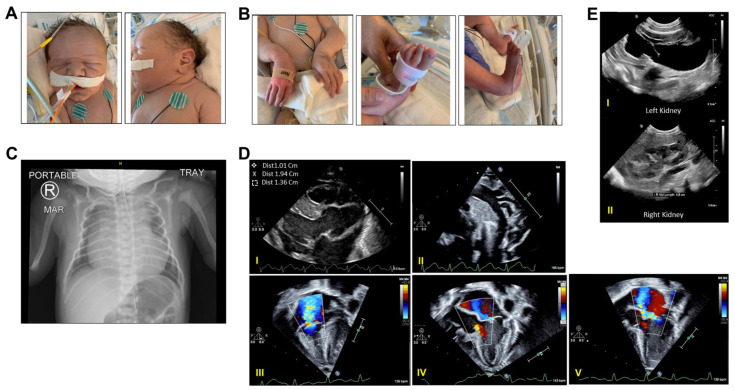
Clinical features of proband A suggesting neonatal Marfan syndrome. (**A**,**B**) Representative photographs of proband A face (**A**) showing and extremities (**B**). (**C**) Representative chest X-ray on day of life 2 (DOL2) showing cardiomegaly and abnormal rib cage. (**D**) Representative echocardiography images on DOL 2. (**I**) Parasternal long axis view showing dilated aortic root (Z-score: +5.7); (**II**) normal left aortic arch with normal pattern branching; (**III**) Color Doppler (CD) interrogation across the tricuspid valve (TV) showing tricuspid regurgitation; (**IV**) CD interrogation across the aortic valve showing mild aortic insufficiency; and (**V**) CD interrogation across the mitral valve (MV) showing mild eccentric MV regurgitation, a feature commonly seen in dysplastic valves. Also, refer to supplemental Video S1. (**E**) Representative kidney ultrasound images on DOL 2. (**I**) Left kidney hydronephrosis SFU grade 3; (**II**) right kidney.

Proband A was born at an outside hospital to a 31-year-old G4P3014 Hispanic mother, weighing 3780 g after a gestation period of 39 weeks. The patient’s prenatal history is significant for premature atrial contractions (PACs) as well as left-sided ureterohydronephrosis. The infant was born via a scheduled repeat low transverse caesarean section and Apgar scores were 3 and 7 at 1 and 5 min of life, respectively. Initially, he was hypotonic with no cry and a heart rate of approximately 100 bpm. Despite initial resuscitation with continuous positive airway pressure (CPAP) up to 100% oxygen supplement, the infant continued to have weak tone, flexed posture, and desaturations with a weak muffled cry. He also had a loud 4/6 holosystolic and a diastolic murmur with a palpable thrill. The remainder of his physical examination was notable for dusky color, skin rash, macrocephaly, folded and creased ears, clenched jaw, long thin fingers, clenched hands, flexed body posture, lack of creases on palms and soles, long thin feet and toes, and abnormal shape of the rib cage (Figure 3A–C).

The infant was admitted to the neonatal intensive care unit (NICU) for cardiorespiratory support. His chest X-ray demonstrated severe cardiomegaly (Figure 3C) and his arterial blood gas analysis (ABG) was significant for severe acidosis with a pH of 7.05 and PaCO_2_ of 106 mmHg. Initial postnatal echocardiogram was notable for moderate right ventricular hypertension with elevated peak tricuspid regurgitation pressure gradient (TRPG) of 49 mmHg and moderate to severe aortic sinus dilatation (Z = +6.5) as well bidirectional atrial shunting through a patent foramen ovale (PFO), small left-to-right shunting through a patent ductus arteriosus (PDA), mild to moderate mitral regurgitation (MR), moderate tricuspid regurgitation, mild aortic regurgitation, and moderate to severe pulmonary sinus dilatation. Despite further treatment with vasopressors, hydrocortisone, surfactant, and broad-spectrum antibiotics, he continued to have persistent severe pulmonary hypertension (PPHN) and hypoxemic hypercapnic respiratory failure with a mean Oxygen Index (OI) of 60 and therefore was transferred to our NICU on day of life (DOL) 3 for further evaluation and management.

**Figure 4:**
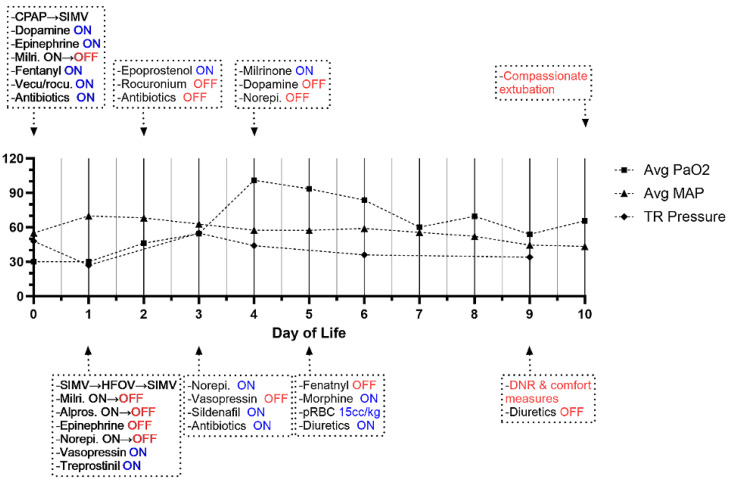
Summary of clinical time course of proband A. Graphs depict clinical parameters throughout the hospital course including the average values of partial pressure of oxygen in the arterial blood (avg PaO2), the average values of mean arterial pressure (Avg MAP), and the peak tricuspid regurgitation (TR) pressure gradient measured on serial echocardiography. *x*-axis: day of life. *y*-axis: measurement absolute value. The text boxes depict important clinical events and treatment interventions over time. CPAP, SIMV, HFOV: different modalities of mechanical respiratory support; pRBC: packed red blood cells.

Despite escalating his respiratory support to mechanical ventilation and starting inhaled nitric oxide to reduce pulmonary pressure, he remained hypoxemic with mean oxygen saturation levels around 85%. A repeat echocardiogram on DOL 3 revealed severe tricuspid regurgitation with prolapse, moderate to severe aortic sinus dilatation (Z = +8.1), severe pulmonary sinus dilatation, as well as new mildly diminished left ventricular systolic shortening (Figure 3D). On the abdomen, renal, and bladder ultrasound, he was found to have grade 3 left hydronephrosis without hydroureter according to the Society of Fetal Urology (SFU) classification (Figure 3E). His PPHN was refractory to prostacyclins (Treprostinil, Epoprostenol), and Sildenafil. Despite maximal medical therapy, he continued to have desaturations and hypotension. Per his parents’ request, his care was redirected toward the patient’s comfort care. On DOL 10, he expired after compassionate extubation per parental request. A summary of the clinical course of proband A is shown in Figure 4.

#### 2.1.2. Case B

**Figure 5:**
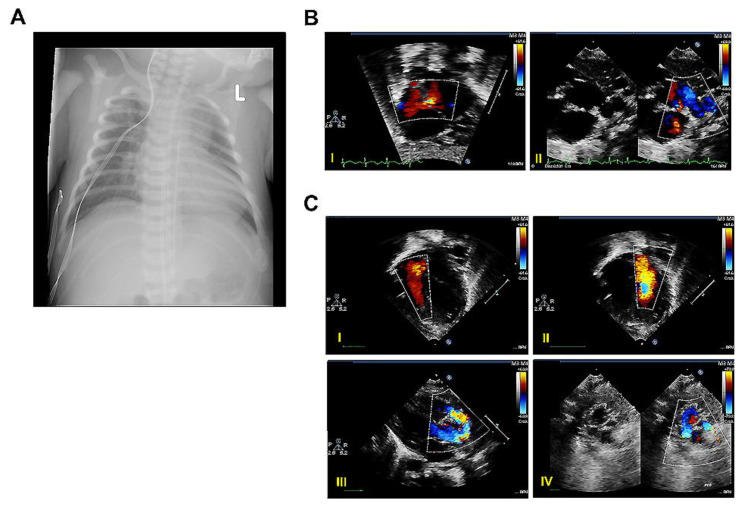
Clinical features of proband B. (**A**) Representative chest X-ray on day of life 0 (DOL 0) showing cardiomegaly. L: Left. (**B**) Representative echocardiography images on DOL 0. (**I**) Color Doppler image across the intra-atrial communication after atrial balloon septoplasty; (**II**) Color Doppler image showing patent ductus arteriosus. (**C**) Representative echocardiography images on DOL 6. (**I**,**II**) Color Doppler images across the tricuspid valve (**I**) and the mitral valve (**II**); (**III**) Color Doppler image of aortic arch after arterial switch operation; (**IV**) B-mode and color doppler images of branch pulmonary arteries after arterial switch operation.

Proband B was born to a 34-year-old G4P2 Hispanic mother, weighing 3410 g after a gestation period of 38 weeks and 3 days. The patient was prenatally diagnosed with Dextro-Transposition of the Great Arteries (D-TGA) with a fetal echocardiogram at 20 weeks of gestation. The infant was born via normal spontaneous vaginal delivery. Apgar scores were 9 and 9 at 1 and 5 min of life, respectively. The infant cried spontaneously at birth and remained notably cyanotic at 5 min of life. In the NICU, he initially presented with paradoxical hypoxemia (i.e., higher lower extremity saturations versus upper extremity saturations). Chest X-ray upon admission was significant for moderate cardiomegaly (Figure 5A) and the initial echocardiogram demonstrated a very small PFO with minimal atrial level shunt. Given persistent hypoxemia in the setting of known D-TGA, the decision was made to pursue a bedside balloon atrial septostomy (BAS) while he was on continuous Prostaglandin E1 (PGE1) infusion to maintain ductal patency with a plan for an atrial switch operation (ASO) in the first week of life. The patient tolerated the septostomy without any complications and a repeat echocardiogram confirmed a large PDA measuring 5.3 mm (Figure 5B). The patient remained at goal oxygen saturations and PGE1 was discontinued on DOL 1 with a repeat echocardiogram on DOL 2 now demonstrating a reduced, moderate PDA measuring 3 mm. He remained hemodynamically stable until DOL 4, when he developed hypoxemic hypercapnic respiratory failure and hypotension requiring mechanical ventilation and vasopressor support, respectively. Additionally, sepsis workup and broad-spectrum antibiotics were initiated on DOL 6; he underwent ASO, PDA ligation, and ASD closure without any complications and was admitted to the pediatric cardiac intensive care unit postoperatively on vasopressors. The echocardiogram demonstrated interval postsurgical changes as well as mild to moderate mitral regurgitation, estimated RVSP of 45 mmHg plus right arterial pressure, and right and left peripheral pulmonary artery stenosis with 45 mmHg and 35 mmHg, respectively (Figure 5C). During the remainder of his hospital course, he was weaned off all cardiopulmonary support without any issues and followed serially with an echocardiogram until being discharged on DOL 20.

The family history of both probands did not reveal consanguinity or other affected family members. There was no history of heart defects, arrhythmias, or sudden deaths.

### 2.2. Genetic Workup

#### 2.2.1. Proband A Cytogenomic Whole-Genome SNP Microarray

The assay was designed to detect alterations to copy number errors (loss or gain) as well as copy-neutral alterations (regions of homozygosity) that indicate an absence or loss of heterozygosity. Copy number variant analysis was performed in accordance with recommendations by the American College of Medical Genetics and Genomics (ACMG). SNP microarray identified a 107 kb interstitial deletion in exons 7–30 of chromosome 15q21.1 of the *FBN1* (Table 2).

#### 2.2.2. Proband B Trio-Whole-Exome Sequencing

Exome variant analysis was performed in accordance with ACMG recommendations. In total, 8 homozygous, including one interval ~6 Mb, variants that were not considered significant or contributary, as well as 212 rare heterozygous protein-altering variants of uncertain clinical significance (VUS’s) were identified across 216 genes. A primary gene list was generated from the Human Gene Mutation Database (HGMD) and Online Mendelian Inheritance in Man (OMIM) using the keywords congenital heart defect, D-TGA, VSD (ventricular septal defect), ASD (atrial septal defect), or PDA (patent ductus arteriosus), and no significant single-nucleotide variants (SNVs) or small deletions and insertions (<10 bp) associated with our patient’s congenital heart defects were detected. However, compound heterozygous c.518C>T and c.8230T>G variants of uncertain clinical significance in the *FBN*2 were identified (Table 3).

## 3. Discussion

We performed clinical phenotyping and genomic analysis of two infants, with nMFS in proband A and D-TGA in proband B, and novel variants in *FBN1* and *FBN2*, respectively.

### 3.1. Neonatal Marfan Syndrome (nMFS)

MFS (MIM:154700) is an inherited autosomal dominant disorder of the connective tissue with major expressions in the skeletal, ocular, and cardiovascular systems and also in the pulmonary system, integument, and dura [18,19,20,21]. Due to the ubiquitous nature of fibrillin microfibrils, MFS often involves multiple organ systems [22]. Hence, FBN1 pathogenic variants are also accountable for diverse albeit overlapping inherited disorders (Supplemental Table S1). Patients with MFS have diverse clinical variability, with nMFS syndrome being the most critical presentation in early childhood [23]. In 2016, 1318 various *FBN1* pathogenic variants linked with MFS were identified; however, only 59 (4.8%) comprising 37 missense mutations (2.8%) were linked with nMFS [23], again indicating that distinct genotype–phenotype associations may potentially dictate the timing and severity of the clinical presentation in the form of neonatal MFS (nMFS) versus MFS [23].

Neonatal Marfan syndrome (nMFS) is the term used to describe patients diagnosed at birth or over the first 3 months of age [24,25,26,27]. Early-onset MFS is caused by de novo variants with no family history and phenotypes expressed in the antenatal, neonatal, or infancy stage preceding that seen in classical MFS [28]. Associated phenotypic features include joint contractures, arachnodactyly, crumpled skin, and ectopia lentis [24,25,26,27]. Congestive heart failure due to mitral and/or tricuspid valve insufficiency is the major cause of death in the first 2 years in nMFS, in contrast to the classic MFS [23,24,25,26]. Chromosomal abnormalities including whole-gene deletion or exon deletions and duplications rarely occurred (3.3%) but have been identified to be on the rise after the adaptation of multiplex ligation-dependent probe technique and array analysis [29].

Like other reported cases, our patient presented with cardinal features of nMFS, including respiratory distress, congenital cardiomegaly, polyvalvular disease, dysmorphic features, and arachnodactyly. In the absence of family history, a clinical diagnosis was made using phenotypic and echocardiographic criteria. The genetic diagnosis was confirmed after infant mortality. Whole-genome SNP microarray was useful in identifying a novel 107 kb interstitial deletion involving chromosome 15 within 15q21.1 spanning exons 7–30 of *FBNI*. This pathogenic variant of *FBN1*, [NM_000138.5], occurred de novo and has not been previously reported. Accordingly, our patient met the criteria for the diagnosis of MFS defined in Ghent nosology with emphasis on the presence of cardiovascular, skeletal, ocular, and skin manifestations in combination with *FBN1* mutation or family history [22,26,30]. Even though pathogenic variants in exons 24–34 are associated with the presence of ectopia lentis in neonatal MFS [31,32], this feature was absent in this patient. Together, the clinical features in combination with the cytogenomic testing provided a strong diagnosis of nMFS. Infant mortality with acute early-onset MFS is 95% during the first year of life with insufficient data on survival into the third and fourth year [28].

The region of interstitial deletion partially overlaps with exon 24–34, which removes a substantial section of the *FBN1* gene, resulting in haploinsufficiency or loss of function of one allele of the gene. Studies have confirmed that a half-normal copy of the FBN-1 protein is not enough to initiate the assembly of microfibrillins [29,33]. Hence, the assumption is that this region plays an essential role in the function of FBN-1 protein, resulting in the earlier onset and severe expressivity of the disease [8,34,35]. The phenotypes and the severity of the disease in our patient could also be attributed to mutation in other regions outside the neonatal region (exon 24–34). About 92% of affected individuals have an affected parent and 25% of probands have novel alteration [30]. Unfortunately, there was no consent for parental testing to evaluate the possible source of the deletion.

Although nMFS is the most severe form of fibrillinopathies, it is poorly diagnosed and genetically/phenotypically different from the classical MFS [23,24,25,26]. Previously, several reports indicated that individuals with nMFS have pathogenic variants in *FBN1* occurring most likely de novo and mainly specific to exons 24–32, referred to as the ‘hotspot’’ or the neonatal region [8,34,35]. Recently, the reason for the pathogenicity of variants in this region has also been attributed to increased proteolytic susceptibility of FBN-1 as well as loss of function for binding of heparin [23], which may affect the arrangement of the microfibrils for their incorporation into polymeric microfibrils and this is a strong indication that the region plays a vital role in elastic microfibril formation [25]. There are also a few reports whereby nMFS could occur due to pathogenic variants outside this region, twice reported in exon 4 and once in exon 21 [35]. Genetic variants in genes including *TGFBR1*, *TGFBR2*, *TGFB2*, *TGFB3*, *SMAD2*, and *SMAD3* result in Loeys–Dietz syndrome and are reported to cause Marfan-like phenotypes and variants in *FBN2* and *COL3A1* result in congenital contractural arachnodactyly and Ehlers–Danlos syndrome type IV [11,36]. Additionally, reports in the literature of patients with MFS phenotype with deletions in chromosome 15q21.1 involving the *FBN1* locus had deletions in other genes [7,29,33,37,38,39] and all these deletions are different from that reported in our patient with deletion resulting in the nMFS.

### 3.2. Congenital Contractual Arachnodactyly (CCA)

Variants in the *FBN2* are associated with autosomal dominant CCA [MIM:121050]. Pathogenic variants identified in *FBN2* assemble in a restricted domain similar to where severe MFS pathogenic variants assemble in *FBN1*, mostly between exons 23 and 32 [14], a region that encodes the cbEGF-like domains, and may occur throughout the gene with a greater number of these pathogenic variants associated with CCA. The pathogenic variant causes a decrease in the amount of FBN-2 protein available to form microfibrils; hence, low levels of microfibril present reduce the elasticity of fibers, resulting in the symptoms exhibited by CCA patients [14,16,40]. Currently, only 91 pathogenic variants in the *FBN2* gene linked with CCA are reported in the literature, as recorded in the Human Genome Mutation Database (HGMD) [41]. In addition, there are no known genotype–phenotype correlations for *FBN2* and congenital cardiac defects such as dextro-transposition of the great arteries (D-TGA) at the time of publication.

Individuals with CCA share many features with nMFS; however, patients with CCA do not present with ectopia lentis or aortic root dilatation, which is the main distinguishing feature [12]. Phenotypes of CCA vary within and between families and do not exhibit geographical or ethnic predilection [42]. Diseases including MFS with aortopathy, talipes, equinovarus, macular degeneration, and scoliosis could also be triggered by a pathogenic variant in *FBN2* [42].

We report a patient with compound heterozygous mutations in the *FBN2* gene. Proband B was prenatally diagnosed with D-TGA based on a fetal echocardiogram at 20 weeks of gestation and required BAS on DOL 0 and subsequent ASO, ASD repair, and PDA ligation on DOL 5. Upon clinical exome sequencing of proband B and his parents, we identified novel compound heterozygous missense, c.518C>T and c.8230T>G (p.Tyr2744Asp), variants in the *FBN2*. Even though variants in *FBN2* are associated with autosomal dominant CCD [43], neither of these variants has previously been reported in the literature in individuals with this condition or with CHDs.

The variant c.518C>T (p.Thr173Ile), inherited from the mother, has previously been observed in the general population at a low frequency, while the variant c.8230T>G (p.Tyr2744Asp), inherited from the father, has not previously been observed in the general population. The two mutated amino acids are not located in any known critical domain. Pathogenicity predictions using two separate software prediction programs, (SIFT (http://sift.jcvi.org/) and PolyPhen (http://genetics.bwh.harvard.edu/pph/)) were not in agreement for both variants. Within the primary gene list and the genes annotated, using HGMD and OMIM, there were no established clinically significant SNVs or small deletions and insertions identified which can elaborate on the primary clinical concerns seen in this patient. The proband’s parents were heterozygous carriers and had no signs of fibrillinopathies associated with the clinical manifestations of the patient, and family history did not reveal consanguinity.

The majority of clinically significant mutations in *FBN2* result in pathogenic missense or splice site variants which usually assemble in the middle section of the gene encoding several amino acids in EGF-like domains by altering critical binding sites (cysteines) and result in an affected protein product [43]. Table 4 provides a summary of reports in the literature on *FBN2* variants that cause CAA and are associated with different heart defects not observed in the patient. In addition, intragenic in-frame deletions in *FBN2* have also been reported to cause a strongly expressed mutant mRNA, implying that the encoded shortened protein is integrated into the ECM and distorts the proper folding of the fibrillin-2 protein [16,43]. In another report, a recurrent intragenic *FBN2* deletion within exons 1–8 was observed in healthy individuals. Hence, this implies that haploinsufficiency may not be the main mechanism of action but rather a dominant-negative effect of the *FBN2* mutant over the wild-type protein [43]. To our knowledge, this is the first report of a patient with compound heterozygous *FBN2* variants who presents with D-TGA not linked with CCA.

## 4. Materials and Methods

### 4.1. Human Studies

All human studies were conducted in accordance with the regulations of the University of California Los Angeles (UCLA) Institutional Review Board (IRB). All subjects provided written informed consent to participate in this study. Electronic medical records, family history, and specimen collection were acquired through the UCLA Congenital Heart Defect (CHD) BioCore following the UCLA-IRB-approved protocols. All specimens were de-identified and coded following acquisition. Prenatal diagnosis was determined based on serial fetal sonography and echocardiography findings. Clinical diagnosis was determined based on clinical features and echocardiography findings.

### 4.2. Cytogenomic SNP Microarray

SNP Microarray was performed for proband A using genomic DNA (gDNA) isolated from peripheral blood monocytes at the UCLA Clinical Genomics Center following clinically validated CLIA (Clinical Laboratory Improvement Amendments)- and CAP (College of American Pathologists)-validated protocols. Whole-genome Single-Nucleotide Polymorphism (SNP) oligonucleotide array was used to assess copy number variations (CNVs), insertions, deletions, duplications, and genomic imbalances in the sample tested. The assay compared proband A’s DNA to an internal reference and to an external reference from 380 normal controls using the Affymetrix Genome-Wide SNP Array CytoScan™ HD (ThermoFisher Scientific, Waltham, MA, USA) for both normalization and comparative analysis. This array platform contains 2.6 million markers for CNV detection, of which 750,000 are genotype SNPs and 1.9 million are non-polymorphic probes, for whole-genome coverage. The analysis was performed using the Affymetrix Chromosome Analysis Suite (ChAS) software, version 3.1.0.15 (r9069).

### 4.3. Trio Whole-Exome Sequencing (WES)

The genomic DNA was extracted from peripheral blood monocytes (PBMCs) at the UCLA Congenital Heart Defects BioCore by using standard methods (Purelink Genomic DNA Mini Kit, Invitrogen, Waltham, MA, USA). Library preparation, sequencing, and data analysis were performed at the CCRD (California Center for Rare Disease) and the UCLA Clinical Genomics Center, using the CLIA-validated protocols. Genomic DNA (3 µg) samples from the proband and parents were subjected to library preparation and exome capture following the Agilent Sure Select Human All Exon 50 Mb (Agilent Technologies, Santa Clara, CA, USA) Illumina Paired-End Sequencing Library Prep Protocol. Sequencing was performed on an Illumina HiSeq4000 (Illumina, San Diego, California, United States) as a 50 bp paired-end run. For each sample, approximately 200 million independent paired reads were generated for an average coverage of 140X of RefSeq protein-coding exons and flanking introns (+/− 2 bp), with at least 95% of these bases covered at ≥10×. The sequences were aligned to the hg19/b37 genome release by using the Novoalign function. PCR duplicates were marked using Picard. The Genome Analysis Toolkit (GATK) [49] was used for indel realignment and base quality recalibration. Both SNVs (single-nucleotide variants) and small INDELs (insertions and deletions) were called using GATK unified genotyper. All variants were annotated using the customized VEP (variant effect predictor) engine from Ensembl. Regions of homozygosity by descent were determined using PLINK. Rare variants with a minor allele frequency of <1% in public databases were retained for further analysis [50].

### 4.4. Variant Analysis

Candidate rare variants were classified based on their zygosity and pattern of inheritance, their location within the gene, conservation scores, population and allele frequencies (ClinVar), predicted consequence at the protein level and structural domains, pathogenicity prediction in silico tools, evidence from functional studies and animal models, and disease spectrum, in accordance with the ACMG/AMP Guidelines for Interpretation of Sequence Variants [51]. All variants were interpreted in the context of the patient’s phenotype. Variants were dismissed if they were predicted to be tolerant (have low impact on protein structure or function) or have been reported in the GnomAD database. Finally, the technical quality of the candidate variants was confirmed using the Integrative Genomics Viewer (IGV) v2.16.0 [52].

## 5. Conclusions

This study reports the first novel interstitial *FBN1* deletion in proband A that occurred de novo. The interstitial deletion has a significant impact on the normal functioning of the protein, resulting in a severe form of nMFS. The phenotypic features seen in our patient signify a genotype–phenotype association in patients with nMFS and also elaborate on the information associated with the clinical features of nMFS. Although we did not observe fibrillinopathy in the form of CCA in proband B, complex congenital heart defects (CCHD) have been reported in *FBN2* mutations. However, the significance of isolated D-TGA in *FBN2* variants in particular remains to be seen, as this conotruncal lesion is typically not associated with any genetic abnormalities or familial inheritance patterns. Therefore, the *FBN2* compound heterozygous variants c.518C>T and c.8230T>G, which are currently classified as VUSs, may represent a possible genotype–phenotype correlation together.

## Figures and Tables

**Table 1:** Clinical features associated with pathogenic variants in *FBN1* and *FBN2*.

Gene	Disorder	MOI	Skeletal Features	Cardiovascular Features	Ophthalmological Features	Other Features
*FBN1*	MFS	AD	Tall stature, arachnodactyly, brachydactyly, scoliosis, pectus, deformities, joint stiffness, contractures, hypermobility of joints, muscle hypoplasia, pes planus, toe walking, muscular build, early-onset carpal tunnel syndrome	Thoracic aortic aneurysms & dissections, mitral/tricuspid valve prolapse	Ectopia lentils Myopia	Pneumothorax, skin striae, long narrow face, malar hypoplasia, micrognathia, retrognathia
*FBN2*	CCA	AD	Arachnodactyly, (kypho)scoliosis, pectus deformities, contractures of knees and ankles, muscle hypoplasia, long, slender fingers & toes, crumpled ears with folded upper helix	Mild cardiovascular involvement		Long narrow face, highly arched palate, micrognathia, crumpled external ears

AD: autosomal dominant; CAA: congenital contractural arachnodactyly; *FBN1*: fibrillin-1; *FBN2*: fibrillin-2; MOI: mode of inheritance; MFS: Marfan syndrome.

**Table 2:** Proband A’s *FBN1* deletion was detected by SNP Microarray.

Gene	Chromosome	Exons Deleted	Interpretation
*FBN1*	15q21	Exon 7-30	107kb interstitial deletion resulting in haploinsufficiency, most likely de novo

**Table 3:** Proband B’s *FBN2* compound heterozygous variants detected by trio-WES.

Gene	Genomic Position (Hg19)	Nucleotide Change	Transcript	Protein Change	Molecular Consequence	Inheritance	Zygosity	Interpretation	Minor Allele Frequency
*FBN2*	5:127863579	c.518C>T	NM_001999.3	Thr173IIe	Missense	Mother	Heterozygous	Uncertain significance	<0.01
*FBN2*	5:127597562	c.8230T>G	NM_001999.3	Tyr2744Asp	Missense	Father	Heterozygous	Uncertain significance	<0.01

**Table 4:** Variants in *FBN2* associated with CCA and congenital heart defects reported in literature.

Gene	Age at Diagnosis	Nucleotide/Protein Change	Effect	Exon/ Intron	Inheritance	Cardiac Abnormalities & Others	Diagnosis	Reference
*FBN2*	34 weeks female	A>T Transversion	Splice variant	Exon 34	NA	Type-B interrupted aortic arch, large ventricular septal defect, moderate atrial septal defect Duodenal atresia intestinal malrotation, single umbilical artery, vertebral anomalies, arachnodactyly, contractures of elbows & knees	CCA	[44]
*FBN2*	5 yrs	G>C Transversion	Splice variant	Exon 31	De novo	Dilated aortic root, prolapsed mitral valve Congenital contractures, arachnodactyly, crumpled helices, scoliosis	CCA	[12]
*FBN2*	At birth	C1239R	NA	Exon 28	NA	Enlarged aortic root Congenital contractures, crumpled ears, arachnodactyly	CCA	[14]
*FBN1&2*	12 yrs female	G>A Transversion	Splice variant	Intron 32	NA	Aortic root dilatation Crumpled appearance to the helix of the ear, contractures of multiple joints, dolichostonomelia, scoliosis, pectus carinatum, striae, highly arched palate	CCA with manifestations of MFS	[45]
*FBN2*	38 weeks 5 days male	NA	NA	NA	NA	Left ventricular noncompaction Arachnodactyly, dolichostenomelia, metatarsus varus, contraction of the elbows & knees, thin extremities & ears with flattered helices, crumpled antihelices	CCA	[46]
*FBN2*	51 yrs male	G>A	Splice variant	Exon 32	NA	Aortic dilatation and/or dissection, Contractures, scoliosis, crumpled appearance to the helices of the ear	CCA	[47]
*FBN2*	NA	A>G	Splice variant	Exon 27	Paternal	Ventricular septal defect Right humerus and bilateral ulnar radius curvature, continuous clenched hands, elongated limbs, finger contracture	CCA	[48]

## Data Availability

The datasets used and analyzed during the current study are available from the corresponding author upon reasonable request. The study was registered in dbGaP under Novel Gene-Environment Regulatory Circuit in Chamber-Specific Growth of Perinatal Heart, Study ID: 45333. The stable dbGaP accession for this study is phs002725.v1. p1.

## Supplementary Materials

The supporting information can be downloaded at: https://www.mdpi.com/article/10.3390/ijms25105469/s1.

## Abbreviations

ABG	Arterial Blood Gas Analysis
ACMG	American College of Medical Genetics
cbEGF	Calcium Binding Epidermal Growth Factor
CCA	Congenital Contractual Arachnodactyly
CPAP	Continuous Positive Airway Pressure
DOL	Day of life
D-TGA	Dextro-Transposition of the Great Arteries
ECM	Extracellular Matrix
EGF	Epidermal Growth Factor
FBN-1	Fibrillin-1
FBN-2	Fibrillin-2
HGMD	Human Gene Mutation Database
LTBP	Latent Transforming Growth Factor β1 Binding Protein
MFS	Marfan Syndrome
MR	Mitral Regurgitation
NICU	Neonatal Intensive Care Unit
nMFS	Neonatal Marfan Syndrome
OI	Oxygen Index
OMIM	Online Mendelian Inheritance in Man
PGE1	Prostaglandin E1
PPHN	Persistent Pulmonary Hypertension
SFU	Society of Fetal Urology
SNP	Single-Nucleotide Polymorphism
SNV	Single-Nucleotide Variants
TGFBR1	Transforming Growth Factor Beta Receptor type 1
TR	Tricuspid Regurgitation
TRPG	Tricuspid Regurgitation Pressure Gradient
UCLA	University of California
VUS	Variant of Uncertain Clinical Significance
WES	Whole-Exome Sequencing
