# Supplementary material for: High-Throughput Genomics Identify Novel FBN1/2 Variants in Severe Neonatal Marfan Syndrome and Congenital Heart Defects
Source: Int J Mol Sci. 2024 May 17;25(10):5469. doi: 10.3390/ijms25105469 (PMC11122089; doi:10.3390/ijms25105469)
Supplement: Supplementary file 1 [file ijms-25-05469-s001.zip › Table S1.pptx]

## Slide 1
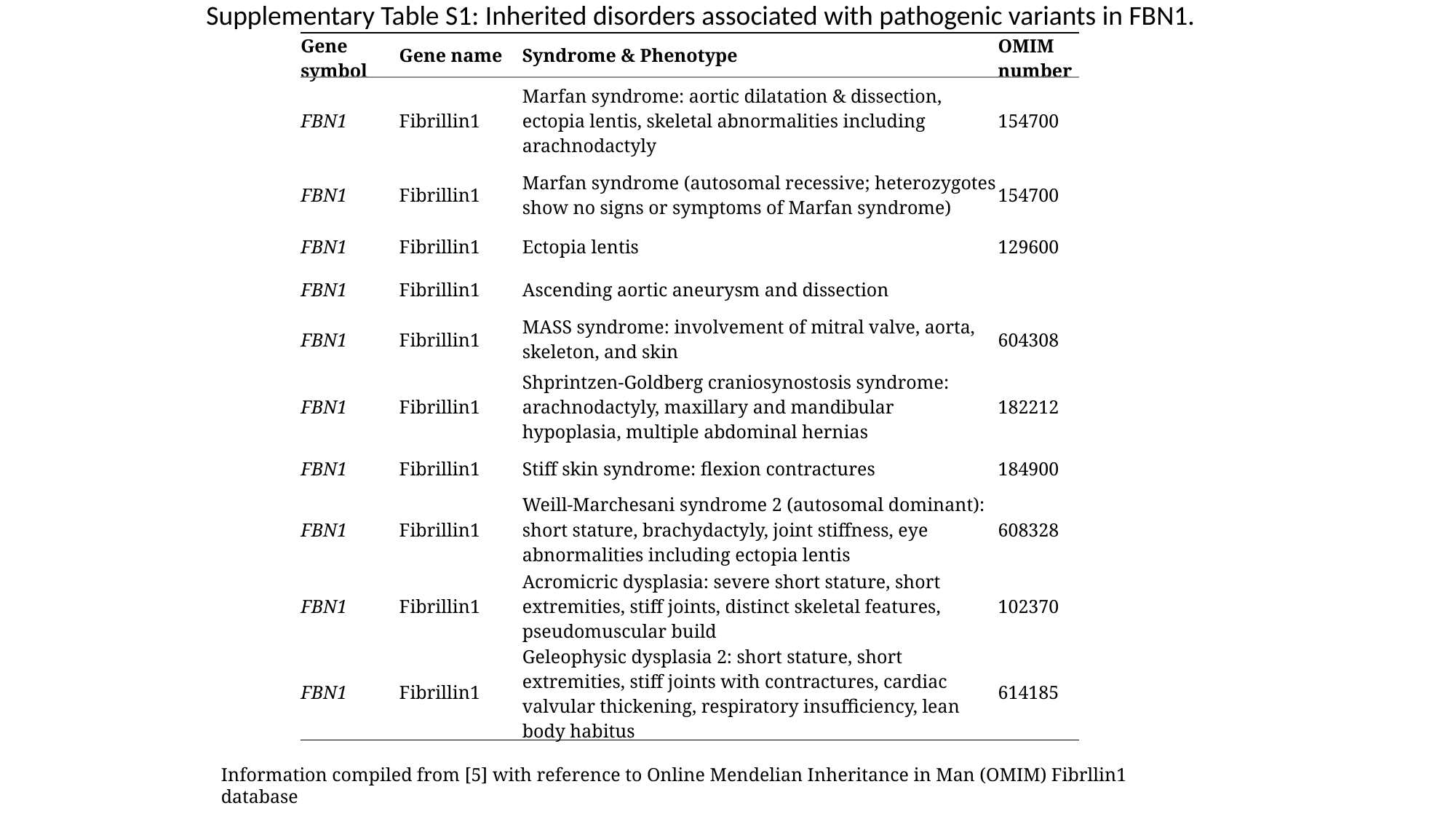

Supplementary Table S1: Inherited disorders associated with pathogenic variants in FBN1.
| Gene symbol | Gene name | Syndrome & Phenotype | OMIM number |
| --- | --- | --- | --- |
| FBN1 | Fibrillin1 | Marfan syndrome: aortic dilatation & dissection, ectopia lentis, skeletal abnormalities including arachnodactyly | 154700 |
| FBN1 | Fibrillin1 | Marfan syndrome (autosomal recessive; heterozygotes show no signs or symptoms of Marfan syndrome) | 154700 |
| FBN1 | Fibrillin1 | Ectopia lentis | 129600 |
| FBN1 | Fibrillin1 | Ascending aortic aneurysm and dissection | |
| FBN1 | Fibrillin1 | MASS syndrome: involvement of mitral valve, aorta, skeleton, and skin | 604308 |
| FBN1 | Fibrillin1 | Shprintzen-Goldberg craniosynostosis syndrome: arachnodactyly, maxillary and mandibular hypoplasia, multiple abdominal hernias | 182212 |
| FBN1 | Fibrillin1 | Stiff skin syndrome: flexion contractures | 184900 |
| FBN1 | Fibrillin1 | Weill-Marchesani syndrome 2 (autosomal dominant): short stature, brachydactyly, joint stiffness, eye abnormalities including ectopia lentis | 608328 |
| FBN1 | Fibrillin1 | Acromicric dysplasia: severe short stature, short extremities, stiff joints, distinct skeletal features, pseudomuscular build | 102370 |
| FBN1 | Fibrillin1 | Geleophysic dysplasia 2: short stature, short extremities, stiff joints with contractures, cardiac valvular thickening, respiratory insufficiency, lean body habitus | 614185 |
Information compiled from [5] with reference to Online Mendelian Inheritance in Man (OMIM) Fibrllin1 database
